# Protective Effect of a Mixture of *Astragalus membranaceus* and *Lithospermum erythrorhizon* Extract against Hepatic Steatosis in High Fat Diet-Induced Nonalcoholic Fatty Liver Disease Mice

**DOI:** 10.1155/2020/8370698

**Published:** 2020-03-19

**Authors:** Doo Jin Choi, Seong Cheol Kim, Gi Eun Park, Bo-Ram Choi, Dae Young Lee, Young-Seob Lee, Sung-Bum Park, Yong Il Park, Geum Soog Kim

**Affiliations:** ^1^Department of Herbal Crop Research, National Institute of Horticultural and Herbal Science, RDA, Eumseong 27709, Republic of Korea; ^2^Department of Biotechnology, The Catholic University of Korea, Bucheon, Gyeonggi-do 14662, Republic of Korea; ^3^Dong IL PharmTec Co. Ltd., Gangnam, Seoul 06296, Republic of Korea

## Abstract

The present study aimed to evaluate the potential synergistic and protective effects of ALM16, a mixture of *Astragalus membranaceus* (AM) and *Lithospermum erythrorhizon* (LE) extract in a ratio of 7 : 3, against hepatic steatosis in high fat diet (HFD)-induced nonalcoholic fatty liver disease (NAFLD) mice. Forty-eight mice were randomly divided into eight groups and orally administered daily for 6 weeks with a normal diet (ND) or high fat diet alone (HFD), HFD with AM (HFD + 100 mg/kg AM extract), HFD with LE (HFD + 100 mg/kg LE extract), HFD with ALM16 (HFD + 50, 100, and 200 mg/kg ALM16), or HFD with MT (HFD + 100 mg/kg Milk thistle extract) as a positive control. ALM16 significantly decreased the body and liver weight, serum and hepatic lipid profiles, including triglyceride (TG), total cholesterol (TC), high-density lipoprotein-cholesterol (HDL), and low-density lipoprotein-cholesterol (LDL), and serum glucose levels, compared to the HFD group. Moreover, ALM16 significantly ameliorated the HFD-induced increased hepatic injury markers, including aspartate aminotransferase (AST), alanine aminotransferase (ALT), alkaline phosphatase (ALP), lactate dehydrogenase (LDH), and gamma-glutamyltransferase (GGT)-1. Furthermore, as compared to the mice fed HFD alone, ALM16 increased the levels of phosphorylated AMP-activated protein kinase (p-AMPK) and acetyl-CoA carboxylase (p-ACC), thereby upregulating the expression of carnitine palmitoyltransferase (CPT)-1 and downregulating the expression of sterol regulatory element-binding protein (SREBP)-1c and fatty acid synthase (FAS). These results demonstrated that ALM16 markedly inhibited HFD-induced hepatic steatosis in NAFLD mice by modulating AMPK and ACC signaling pathways, and may be more effective than the single extracts of AM or LE.

## 1. Introduction

Nonalcoholic fatty liver disease (NAFLD) is one of the common chronic liver diseases in metabolic syndrome, resulting from the hepatic lipid accumulation of >5% of liver weight [1]. The liver is the major contributor to whole-body lipogenesis, gluconeogenesis, and cholesterol metabolism [2, 3]. The development of NAFLD is characterized by abnormal liver function and can progress to more severe liver diseases such as nonalcoholic steatohepatitis (NASH), fibrosis, and cirrhosis [4, 5]. NAFLD has become a critical health concern worldwide owing to its increased morbidity and mortality. However, there are no approved agents currently available for the treatment of NAFLD [6]. NAFLD patients generally require long-term treatment, which can potentially induce serious or diverse side effects. Therefore, the identification of potential therapeutic compounds with better biological efficacy and safety, and fewer side effects is imperative [7].

Until now, the pathogenesis of NAFLD remained poorly understood; the ‘two-hit' concept is generally recognized as the most important theory. The ‘first hit' is the excessive accumulation of triglycerides (TG) in hepatocytes, and ‘second hits' are associated with increased inflammatory adipokines/cytokines, such as adiponectin, tumor necrosis factor (TNF)-*α* and interleukin (IL)-6, oxidative stress, and mitochondrial impairment [8, 9]. Accordingly, therapeutic strategies for NAFLD have focused on identifying potential agents that can exert effects to prevent hepatic steatosis and inflammation.

The AMP-activated protein kinase (AMPK) signaling pathway plays an important role in maintaining cellular energy metabolism [10]. AMPK, involved in mediating lipogenesis and fatty acid oxidation in the liver, has become a major target for the treatment and/or prevention of NAFLD [11]. Activation of AMPK mediates energy metabolism through the suppression of acetyl-CoA carboxylase (ACC) and fatty acid synthase (FAS) expression, via the decreased transcriptional activation of the sterol regulatory element-binding protein (SREBP)-1c [12]. Inactivation of ACC by activated AMPK leads to the reduced synthesis of malonyl-CoA, which decreases fatty acid synthesis and increases mitochondrial fatty acid oxidation via regulation of carnitine palmitoyltransferase (CPT)-1 [13].

Recently, many medicinal herbs have gained attention as potential therapeutic agents in the treatment of various human diseases. In traditional oriental medicine, major medicinal herbs are frequently used in combination with other herbs for the treatment of chronic or complex diseases, including liver disease [14]. Herb-herb combinations have long been used in Oriental medicine for thousands of years, yet scientific evidence of their therapeutic benefits was lacking. Accordingly, scientific interest to study the combination therapy for the treatment of diseases is increasing since scientific literature demonstrating evidence of enhanced efficacy, thus called ‘synergistic effects', for certain diseases when treated in a combination of different herbs than treated individually, is now piling up [15–17].

*Astragalus membranaceus* (AM), called Hwangki in Korea, is an edible herb widely consumed for various medical symptoms owing to its diverse pharmaceutical activities, including modulation of the immune system, and antitumor properties [18, 19]. AM is enriched with isoflavonoids, triterpene saponins, and polysaccharides [20]. Among them, calycosin and calycosin-7-*O*-*β*-D-glucoside are used as chemical markers for the comprehensive quality evaluation of AM, due to the presence of various biological activities [21, 22]. Previously, calycosin was shown to exert hepatoprotective effects in acute liver injury and chronic liver diseases [23, 24]. The AM extract has been frequently consumed in combination with various other herbal mixtures in traditional folk medicines in Korea due to its many health-beneficial activities and synergistic effects under different pathological conditions, *in vitro* and *in vivo* [25, 26].

*Lithospermum erythrorhizon* (LE) has also been used in traditional medicine for its various pharmacological effects. LE contains naphthoquinones, including shikonin and its derivatives, identified as the major phytochemicals [27]. Recent studies have reported the various biological activities of LE, including anti-inflammatory, antiobesity, and antilipogenic activities [28–30]. Especially, Gwon et al. reported that ethanol extract of LE, which contains acetylshikonin as the main compound, exerted antiobesity effect through inhibition of lipogenic and adipogenic gene expression [29]. They also showed that the same sample prevented the hepatic fat accumulation, although detailed underlying action mechanism on its inhibitory activity against hepatic fat accumulation was not extensively addressed [29]. Like AM, LE has also garnered much attention as a complementary herb that can act simultaneously on various targets in diseased states [31, 32]. A study by Roh [33] reported that Gangjihwan, a polyherbal drug composed of LE as the main ingredient, demonstrated a beneficial effect in NAFLD mice via the regulation of the genes involved in lipid metabolism and inflammation.

In our previous study, we selected the effective ratio (7 : 3, w/w) of a combination of AM and LE extracts to measure the activities of matrix metalloproteinases (MMP) −1, −3, and −13 [34]. Subsequently, we reported that a mixture of these two herbal extracts at the ratio of 7 : 3 (AM : LE, 7 : 3, w/w; referred to as ALM16), as a new herbal composition, has an antiosteoarthritis activity based on synergistic action [35]. However, despite the reported health benefits and potential synergistic actions associated with these two herbs, the hepatoprotective and synergistic effects of a mixture of AM and LE have not been explored yet. In this regard, to extend the possibility of the combined mixture of AM and LE as a new and/or improved therapeutic modality to other health problems beyond its antiosteoarthritis activity, thereby increasing the medicinal applicability of these two herbs, this study was aimed to evaluate a possible positive effect of ALM16 against NAFLD. For this purpose, this study was designed to investigate the protective effects of ALM16 on hepatic steatosis using the high fat diet (HFD)-induced NAFLD mouse model. The underlying mechanisms of the antihepatic steatosis activity in the NAFLD model are also discussed. In addition, we examined whether the combined treatment with AM and LE, rather than the use of individual herb alone, could possibly show an enhanced effect against NAFLD. For comparison, *Silybum marianum* (milk thistle) extract (MT), known for its antisteatosis activity, was used as a positive control in the *in vivo* experiments [36].

## 2. Materials and Methods

### 2.1. Ethics Statement

All mice were treated in accordance with the Guide for the Care and Use of Laboratory Animals, as approved by the Institutional Animal Care and Use Committee of the Catholic University of Korea (Proved No. 018–011), and all efforts have been made to minimize the number of animals used.

### 2.2. Preparation of Herbal Mixture (ALM16)

AM and LE were collected from Jecheon, Chungcheongbuk-do, Korea. AM and LE, which were assigned voucher specimen number (MPS005087 and MPS004961, respectively), were authenticated by Dr. Jeong Hoon Lee, Ph.D., from the National Institute of Horticultural and Herbal Science, Rural Development Administration, Korea. The mixture of AM and LE extracts (ALM16) was prepared according to the method described in our previous study [35]. Briefly, the dried roots of AM and LE were extracted with 50% and 70% aqueous fermented ethanol at 80°C for 4 h, respectively. Individual AM and LE dried extracts were mixed well in a ratio of 7 : 3 (w/w), to prepare the ALM16. The appropriate amounts of AM, LE, and ALM16 were dissolved in phosphate-buffered saline (PBS) for the experiments, and stored at −20°C.

### 2.3. Animal Experimental Design

Male C57BL/6 mice (5 weeks old), purchased from the ORIENT BIO INC (Gyeonggi-do, Korea), were housed in the specific pathogen-free (SPF) facility, and maintained under controlled temperatures (22–23°C), on a 12/12 h light-dark cycle in cages. After the acclimatization period, 48 male C57BL/6 mice (initially weighing approximately 19.3–20.3 g) were randomly divided into eight groups (*n* = 6) as follows: (1) normal diet group (ND, 16% calories from fat), (2) high fat diet group (HFD, 60% calories from fat), (3) HFD + 100 mg/kg AM extract group (AM), (4) HFD + 100 mg/kg LE extract (LE), (5) HFD + 50 mg/kg ALM16 (ALM16-L), (6) HFD + 100 mg/kg ALM16 (ALM16-M), (7) HFD + 200 mg/kg ALM16 (ALM16-H), and (8) HFD + 100 mg/kg Milk thistle extract group (MT, as a positive control group). After 7 weeks of HFD-induced NAFLD, all samples were dissolved in PBS and orally administered daily for 6 weeks. The groups fed with ND or HFD only were administered PBS. Body weights and food intake were measured every week. At the end of the experimental period, all mice underwent fasting for 12 h and were then anesthetized using a mixture of Zoletil (50 mg/ml, Virbac, Carros, France)/Rompun (23.32 mg/ml, Bayer, Germany) (1 : 3). Blood samples were collected by cardiac puncture. The liver tissues were then collected and weighed.

### 2.4. Serum and Tissue Biochemical Analysis

Serum levels of alanine aminotransferase (ALT), aspartate aminotransferase (AST), alkaline phosphatase (ALP), triglyceride (TG), total cholesterol (TC), high-density lipoprotein-cholesterol (HDL), low-density lipoprotein-cholesterol (LDL), and glucose were measured using an AU680 Automatic Analyzer (Beckman coulter, CA, USA), and lactate dehydrogenase (LDH) was measured using a commercial LDH assay kit (Sigma, St. Louis, MO, USA). Lipid in the liver tissue was extracted using modified methods, as previously described [37]. Briefly, stored liver tissues were homogenized in chloroform-methanol-distilled water (2 : 1 : 1, v/v/v) using a homogenizer for 1 min, and then centrifuged at 13,000 rpm for 15 min at 4°C. The solvent (chloroform) phase obtained from the extraction was dried and dissolved in methanol, and then the hepatic TG levels were measured using a triglyceride assay kit (Cayman Chemical, Ann Arbor, MI, USA).

### 2.5. Western Blot Analysis

The liver tissues were homogenized using a tissue grinder (Nippon genetic, Germany), with 500 *μ*l of the lysis buffer for 30 s. The supernatant was then obtained by repeated centrifugation for 15 min at 13,000 rpm, at 4°C. The protein concentrations of lysates were determined using the Bio-Rad protein assay reagent (Bio-Rad, Hercules, GA, USA). Equal amounts of protein were separated on 6–13% sodium dodecyl sulfate (SDS)-polyacrylamide gel electrophoresis (PAGE) and transferred onto a nitrocellulose membrane. Membranes were blocked using a 5% nonfat milk blocking solution for 2 h. The membranes were incubated with the primary antibodies against AMP-activated protein kinase (AMPK), phospho-AMPK, acetyl-CoA carboxylase (ACC), phospho-ACC, FAS (Cell Signaling Technology, Beverly, MA, USA), CPT-1, SREBP-1c (Santa Cruz Biotechnology, Dallas, TX, USA) and gamma-glutamyltransferase (GGT)-1 (Abcam, Cambridge, MA, UK) at 4°C overnight, washed 3 times with Tris-buffered saline containing Tween 20 (TBST) for 5 min and then incubated with HRP-conjugated secondary antibodies for 1 h. Immunoreactive bands were then developed using a Chemiluminescence detection reagent (Pierce Biotechnology, Rockford, IL) and exposed to X-ray film (Konica, Tokyo, Japan). *β*-Actin was used as a loading control. The bands were quantified by densitometric analysis with Image J software.

### 2.6. Histopathological Analysis

To investigate the effect of ALM16 on hepatic steatosis induced by HFD in mice, liver tissues were examined by histopathological analysis. The liver tissue was fixed in 10% buffered formalin for 48 h, embedded in paraffin, and sectioned into 3-*μ*m-thick sections. The liver sections were stained with hematoxylin and eosin (H&E) for histological analysis. Images were acquired using a light microscope (Olympus, Tokyo, Japan). Histopathological examinations were performed by a board certified toxicologic pathologist, blinded to the treatment strategy.

### 2.7. Statistical Analysis

All values are expressed as mean ± standard error of mean (SEM). The results were evaluated using one-way ANOVA with Dunnett's multiple comparison test using Graph-Pad Prism® software version 5.01 (San Diego, CA, USA). The differences between the data from the HFD and sample-treated groups were considered statistically significant when *P* < 0.05.

## 3. Results

### 3.1. ALM16 Inhibits Hepatic Steatosis in the Liver of HFD-Fed Mice

**Figure 1:**
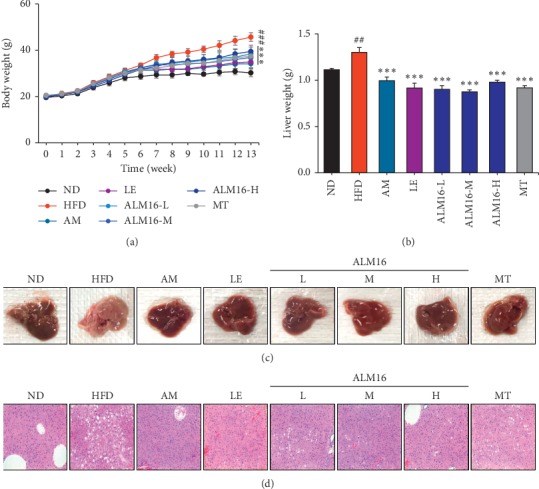
Effects of ALM16 and individual extracts (AM and LE) on hepatic steatosis in the liver of HFD-fed mice. (a) Body weight and (b) Liver weight (*n* = 6). (c) Representative macroscopic images of the liver. (d) Representative histopathological images of the liver tissue, stained with hematoxylin and eosin (H&E), of HFD-fed mice (magnification, 200x). Data are presented as mean ± standard error of the mean (SEM). ^#^*P* < 0.05, ^##^*P* < 0.01, and ^###^*P* < 0.001 vs ND group; ^*∗*^*P* < 0.05, ^*∗∗*^*P* < 0.01, and ^*∗∗∗*^*P* < 0.001 vs HFD group. ND: normal diet; HFD: high fat diet; AM: HFD + AM extract (100 mg/kg); LE: HFD + LE extract (100 mg/kg); ALM16-L: HFD + ALM16 (50 mg/kg); ALM16-M: HFD + ALM16 (100 mg/kg); ALM16-H: HFD + ALM16 (200 mg/kg); MT: HFD + milk thistle extract (100 mg/kg).

The body and liver weights in mice are shown in Figures 1(a) and 1(b). Both body (*P* < 0.001) and liver weights (*P* < 0.01) significantly increased in the HFD group, compared to the ND group. However, the body weight (*P* < 0.001) and liver weight (*P* < 0.001) in ALM16-treated groups (50, 100 and 200 mg/kg) significantly decreased by 30.8%, 32.8%, and 24.8%, respectively, compared to the HFD group. During the experiment period, no significant differences in food intake were observed between the HFD group (2.8 g/day/mice) and all sample-treated groups (2.5–2.8 g/day/mice, Supplementary [Supplementary-material supplementary-material-1]). AM, LE, and MT also significantly inhibited liver weight gain by 23.5% (*P* < 0.001), 29.7% (*P* < 0.001), and 29.4% (*P* < 0.001), respectively, compared with the HFD group. Although ALM16 demonstrated similar inhibitory effects on body weight gain, it inhibited liver weight gain more effectively compared with that in AM, LE, or MT-treated groups. In addition, the liver tissue of the HFD group was pale and yellow in appearance with severe accumulation of lipid droplets compared to that of the ND group which showed normal hepatic conditions (Figures 1(c) and 1(d)). However, a drastic decrease in hepatic lipid deposition was observed in all sample-treated groups (Figures 1(c) and 1(d)). The ALM16 (100 mg/kg) group demonstrated mild steatosis in the liver tissue, similar to the AM and LE-treated groups, but was more effective than MT (100 mg/kg) group (Figures 1(c) and 1(d)). In the ALM16 groups, although no dose-dependent differences were observed, the group administered 100 mg/kg showed greater effective amelioration of hepatic steatosis than those groups treated with 50 and 200 mg/kg ALM16. These results suggested that ALM16 (100 mg/kg) has a strong inhibitory effect against HFD-induced hepatic steatosis.

### 3.2. Effects of ALM16 on Liver Damage Markers in HFD-Fed Mice

**Figure 2:**
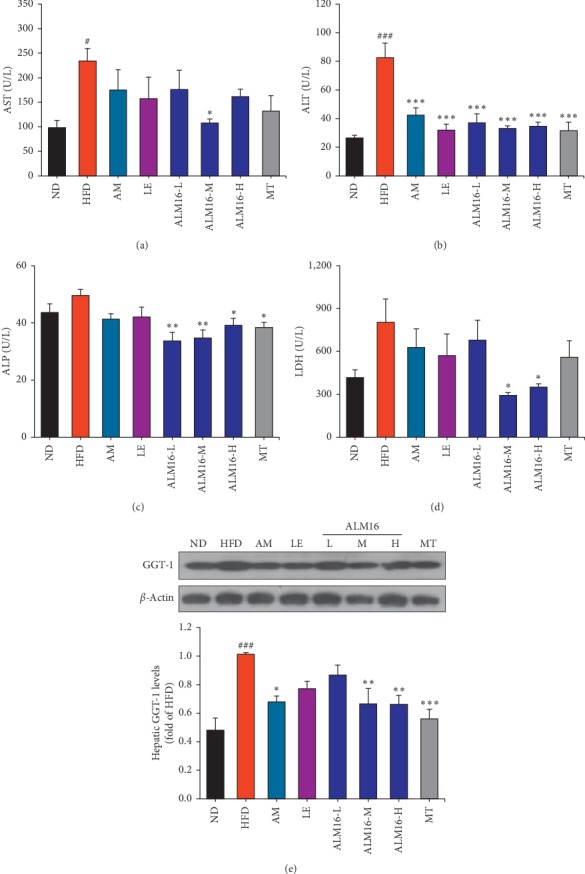
Effects of ALM16 and individual extracts (AM and LE) on biochemical parameters in the serum of NAFLD mice. Serum levels of (a) AST, (b) ALT, (c) ALP, and (d) LDH in HFD-fed mice (*n* = 5). (e) Protein expression levels of hepatic GGT-1 (*n* = 4). Data are expressed as mean ± standard error of the mean (SEM). ^#^*P* < 0.05, ^##^*P* < 0.01 and ^###^*P* < 0.001 vs ND group; ^*∗*^*P* < 0.05, ^*∗∗*^*P* < 0.01, and ^*∗∗∗*^*P* < 0.001 vs HFD group. ND: normal diet; HFD: high fat diet; AM: HFD + AM extract (100 mg/kg); LE: HFD + LE extract (100 mg/kg); ALM16-L: HFD + ALM16 (50 mg/kg); ALM16-M: HFD + ALM16 (100 mg/kg); ALM16-H: HFD + ALM16 (200 mg/kg); MT: HFD + milk thistle extract (100 mg/kg).

The hepatic injury caused by HFD and the protective effects of ALM16 were assessed by measuring the levels of hepatic biochemical markers in the serum and liver. As shown in Figures 2(a)–2(d), the serum levels of AST (*P* < 0.05), ALT (*P* < 0.001), ALP, and LDH increased in the HFD group, compared to those in ND group, indicating that hepatic damage was induced by HFD administration. However, these HFD-induced elevated serum levels of AST, ALT, ALP, and LDH were markedly reduced following the administration of ALM16 (100 mg/kg) by 54.0% (*P* < 0.05), 59.9% (*P* < 0.001), 30.0% (*P* < 0.01), and 63.7% (*P* < 0.05), respectively, compared with the levels in the HFD group. Furthermore, serum levels of these markers were much lower in the ALM16-treated group, especially the ALM16-M (100 mg/kg)-treated group, compared to the groups treated with AM, LE, or MT alone. The results demonstrated that the HFD-induced hepatic damage is more attenuated by ALM16 than in individual extracts (AM or LE). Additionally, the hepatic GGT-1 level significantly increased in the HFD group compared with that in the ND group (Figure 2(e), (*P* < 0.001). In comparison with the hepatic GGT-1 level of HFD group, AM and LE decreased the hepatic GGT-1 level by 33.1% (*P* < 0.05) and 23.9%, respectively, and ALM16 (50, 100 and 200 mg/kg) treatment also significantly reduced the GGT-1 level by 14.6%, 34.4% (*P* < 0.01), and 34.8% (*P* < 0.01), respectively. These results clearly suggest that ALM16 ameliorates the HFD-induced liver damage and is more effective than AM or LE extract alone.

### 3.3. Effects of ALM16 on Serum and Liver Lipids in HFD-Fed Mice

**Figure 3:**
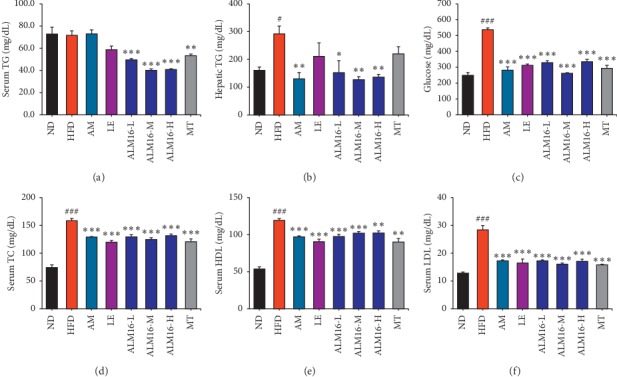
Effects of ALM16 and individual extracts (AM and LE) on serum lipids and glucose levels in HFD-fed mice. (a) Serum TG and (b) hepatic TG levels. (c) Serum glucose levels. Serum levels of (d) TC, (e) HDL, and (f) LDL cholesterol in HFD-fed mice (*n* = 5). Values are presented as mean ± standard error of the mean (SEM). ^#^*P* < 0.05, ^##^*P* < 0.01, and ^###^*P* < 0.001 vs ND group; ^*∗*^*P* < 0.05, ^*∗∗*^*P* < 0.01, and ^*∗∗∗*^*P* < 0.001 vs HFD group. ND: normal diet; HFD: high fat diet; AM: HFD + AM extract (100 mg/kg); LE: HFD + LE extract (100 mg/kg); ALM16-L: HFD + ALM16 (50 mg/kg); ALM16-M: HFD + ALM16 (100 mg/kg); ALM16-H: HFD + ALM16 (200 mg/kg); MT: HFD + milk thistle extract (100 mg/kg).

As shown in Figure 3, ALM16 (100 mg/kg) significantly reduced the serum and hepatic TG (Figures 3(a) and 3(b)), serum TC (Figure 3(d)), HDL (Figure 3(e)), and LDL (Figure 3(f)) levels by 44.1%, 56.5%, 21.6%, 14.5%, and 43.7%, respectively, compared to the HFD group. Moreover, the elevated serum glucose level in the HFD group significantly decreased in the ALM16 (100 mg/kg)-treated group by 51.2% compared with the HFD group (Figure 3(c)). Although the serum TC, HDL, and LDL levels, in ALM16 (100 mg/kg) group, were similar to the other sample groups, the serum and hepatic TG and glucose levels were more significantly reduced by ALM16 compared to the other sample groups.

### 3.4. ALM16 Increases Phosphorylation of AMPK and ACC in the Liver of HFD-Fed Mice

**Figure 4:**
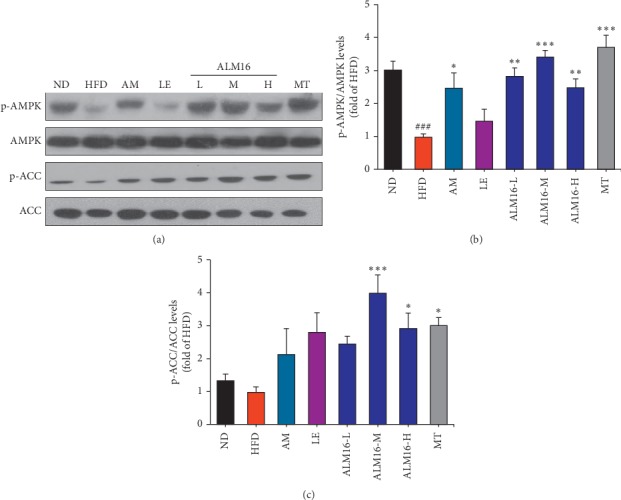
Effects of ALM16 and individual extracts (AM and LE) on hepatic AMPK and ACC phosphorylation in HFD-fed mice. (a) Western blot analysis of AMPK, p-AMPK, ACC, and p-ACC in the liver tissues of HFD-fed mice. Bands were quantified as the ratio of (b) p-AMPK/AMPK and (c) p-ACC/ACC, respectively, with values normalized to the HFD group (*n* = 4). Values are presented as mean ± standard error of the mean (SEM). ^#^*P* < 0.05, ^##^*P* < 0.01, and ^###^*P* < 0.001 vs ND group; ^*∗*^*P* < 0.05, ^*∗∗*^*P* < 0.01, and ^*∗∗∗*^*P* < 0.001 vs HFD group. ND: normal diet; HFD: high fat diet; AM: HFD + AM extract (100 mg/kg); LE: HFD + LE extract (100 mg/kg); ALM16-L: HFD + ALM16 (50 mg/kg); ALM16-M: HFD + ALM16 (100 mg/kg); ALM16-H: HFD + ALM16 (200 mg/kg); MT: HFD + milk thistle extract (100 mg/kg).

As presented in Figure 4(a) and 4(b), phosphorylated AMPK (p-AMPK)/AMPK levels decreased in the HFD group compared with the ND group. The AM group demonstrated a 2.5-fold (*P* < 0.05) increase in the p-AMPK/AMPK levels compared with that of the HFD group (1.0-fold), but the LE-treated group showed no significant difference. ALM16 (50, 100 and 200 mg/kg) groups significantly increased p-AMPK/AMPK levels by 2.8-fold (*P* < 0.01), 3.4-fold (*P* < 0.001) and 2.5-fold (*P* < 0.01), respectively, compared to the HFD group. Moreover, compared to the HFD group, levels of phosphorylated ACC (p-ACC)/ACC increased in the AM and LE groups with no significant difference (Figure 4(c)). However, ALM16 (100 mg/kg) significantly increased the p-ACC/ACC levels by 4.2-fold (*P* < 0.001), compared to the HFD group. These results showed that ALM16 has a higher ability to activate the AMPK/ACC pathway than individual extract, possibly based on synergistic action. Thus, administration of the combined mixture of these two herbal extracts (AM : LE, 7 : 3, w/w; referred to as ALM16) was shown to be more effective against AMPK/ACC pathway-related NAFLD, compared with the results from individual herb (AM or LE) treatment.

### 3.5. Effects of ALM16 on the Protein Expression of CPT-1, SREBP-1c, and FAS

**Figure 5:**
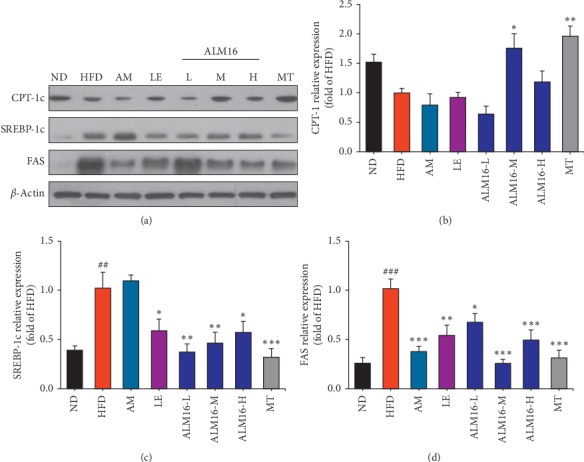
Effects of ALM16 and individual extracts (AM and LE) on the protein expression in hepatic lipid metabolism, CPT-1, SREBP-1c, and FAS, in HFD-fed mice. (a) Western blot analysis of CPT-1, SREBP-1c, and FAS in the liver tissues of HFD-fed mice. The protein expression levels of (b) CPT-1, (c) SREBP-1c, and (d) FAS normalized to *β*-actin relative to HFD-fed mice (*n* = 4). Values are presented as mean ± standard error of the mean (SEM). ^#^*P* < 0.05, ^##^*P* < 0.01, and ^###^*P* < 0.001 vs. ND group; ^*∗*^*P* < 0.05, ^*∗∗*^*P* < 0.01, and ^*∗∗∗*^*P* < 0.001 vs. HFD group. ND: normal diet; HFD: high fat diet; AM, HFD + AM extract (100 mg/kg); LE: HFD + LE extract (100 mg/kg); ALM16-L: HFD + ALM16 (50 mg/kg); ALM16-M: HFD + ALM16 (100 mg/kg); ALM16-H: HFD + ALM16 (200 mg/kg); MT: HFD + milk thistle extract (100 mg/kg).

The protein expressions of CPT-1, SREBP-1c, and FAS in the liver of HFD-fed mice are shown in Figure 5(a). In the HFD group, the protein expression of CPT-1 decreased, and the protein levels of SREBP-1c (*P* < 0.01) and FAS (*P* < 0.001) significantly increased, compared to the ND group. The reduced CPT-1 level in the HFD-fed mice was increased in the ALM16 (*P* < 0.05) groups, but the AM and LE group showed no significant change (Figure 5(b)). Compared to the HFD group, LE and ALM16 (100 mg/kg) groups significantly suppressed the protein expression of SREBP-1c by 42.5% (*P* < 0.05) and 54.7% (*P* < 0.01), respectively, but the AM group revealed no significant change (Figure 5(c)). Furthermore, as shown in Figure 5(d), all samples (AM, LE, and ALM16) at a dose of 100 mg/kg significantly decreased the FAS protein expression by 63.0% (*P* < 0.001), 46.8% (*P* < 0.01), and 74.7% (*P* < 0.001), respectively. MT (100 mg/kg) increased the protein expression of CPT-1 by 2.0-fold (*P* < 0.01) and suppressed the protein expression of SREBP-1c and FAS by 68.8% (*P* < 0.001) and 69.4% (*P* < 0.001), respectively, compared to the HFD group.

## 4. Discussion

Nonalcoholic fatty liver disease (NAFLD) is defined as impaired lipid and glucose metabolism caused by abnormal liver function, such as hepatic steatosis presented as excessive lipid accumulation in the liver [8]. However, no clinically effective therapeutic agents for the treatment of NAFLD are currently available [38]. Recent studies have reported that many herbal medicines for the treatment of NAFLD tend to use multiple herbs in complex formulations, owing to multitargeting and multifunctional properties, complementary interactions, and synergistic effects demonstrating better or enhanced efficacies over treatment with individual herbs [39–41].

In our previous study, we demonstrated that ALM16, mainly composed of calycosin, calycosin-7-*O*-*β*-D-glucoside, and lithospermic acid as index compounds, exerts a synergistic chondroprotective effect in a monosodium iodoacetate (MIA)-induced osteoarthritis rat model [35]. A previous study by Duan [24] reported that calycosin, a major component of Astragali radix, ameliorates HFD-induced NAFLD by inhibiting gluconeogenesis through the modulation of Farnesoid *X* receptor (FXR) activation. In addition, shikonin, which is one of the naphthoquinones in LE, suppresses lipogenesis by increasing the phosphorylated AMPK in hepatocytes [30]. Based on these findings, we postulated that ALM16 would possess a certain level of cartilage protective effect and could exert multiple biological activities under various pathological conditions. To verify this hypothesis, the present study investigated the potential beneficial and synergistic effects of ALM16 on NAFLD using the HFD-fed mouse model. HFD feeding reportedly increases body and liver weights and elevates the levels of lipid parameters and hepatic injury markers in mice, resulting in the development of hepatic steatosis and injury [42]. Thus, rodent NAFLD models induced with dietary interventions (high fat, carbohydrate, and/or cholesterol diets) have generally been accepted as essential tools for research in NAFLD, as they mimic the pathophysiological and phenotypical symptoms in human NAFLD [43].

In this study, the NAFLD mouse model was established by feeding the HFD for 7 weeks. The resulting NAFLD mice reported increased body and liver weights and elevated levels of serum and hepatic lipid profiles. In HFD-fed mice, our results demonstrated that these increased body and liver weights and elevated levels of serum and hepatic lipid parameters were significantly decreased following ALM16 administration. In comparison to AM and LE, ALM16 (100 mg/kg)-treated group demonstrated relatively lower serum TG levels, whereas similar effects with AM or LE, on the hepatic TG, serum TC, HDL, and LDL levels, were observed. HFD feeding is also known to be implicated in the impairment of hepatic glucose metabolism by reducing the ability of insulin to inhibit gluconeogenesis in the liver [44, 45]. Previous studies have reported that HFD feeding in rodents elevates serum glucose levels due to an increase in the hepatic insulin resistance [46, 47]. In this study, ALM16 (100 mg/kg) also effectively reversed the highly elevated serum glucose levels in HFD-fed mice. These results indicated that ALM16 could improve hepatic lipid profiles and glucose metabolism in HFD-fed mice.

It is well known that the serum levels of AST, ALT, ALP, and LDH, which are major enzymes presented in hepatocytes, increase following hepatocellular injury [48]. Among them, especially ALT, is a sensitive biomarker as an indicator associated with NAFLD [49]. In many clinical studies on NAFLD, an increased level of ALT has been considered as an independent predictor [50]. It is also generally accepted that GGT, which is an enzyme abundant in the liver, is elevated as a result of obesity and liver damage, and thus regarded as one of the predictors of liver mortality [51]. ALM16 administration significantly decreased the elevated serum AST, ALT, ALP, and LDH levels caused by HFD and reduced GGT-1 levels in the liver tissue of HFD-fed mice. In particular, ALM16 (100 mg/kg) was more effective on the serum levels of AST, ALP, and LDH than AM or LE, although the effects on the serum ALT and hepatic GGT-1 levels were similar to AM or LE groups. Moreover, histopathological analysis of liver tissues with H&E showed that ALM16 administration markedly attenuated the excessive formation and accumulation of lipid droplets in hepatocytes. Hepatic steatosis, the first hallmark of NAFLD, is known to be characterized by excessive accumulation of lipid in hepatocytes [52]. Several previous studies have provided evidence regarding the hepatoprotective properties against HFD-induced NAFLD by reducing the liver damage markers and inhibiting the accumulation of lipid droplets in the liver tissues of HFD-fed rodents [53, 54]. In parallel, our results demonstrated that ALM16 can exert protective effects and synergistic action against hepatic steatosis and liver injury in HFD-fed mice.

We further investigated the molecular mechanism by which ALM16 provides a protective effect against hepatic steatosis by monitoring the lipid metabolism (lipogenesis and fatty acid oxidation) associated proteins and AMPK and ACC pathways. AMPK is considered as a major therapeutic target for the treatment of NAFLD as it plays an important role in the control of lipid metabolism by regulating lipogenesis and fatty acid oxidation in the liver [12]. The activation of AMPK induces the inactivation of ACC, which decreases the conversion of acetyl-CoA to malonyl-CoA, thereby increasing CPT-1 expression [55]. Increased CPT-1 expression stimulates fatty acid oxidation, and consequently reduces hepatic lipid accumulation [56]. Our study revealed that ALM16 markedly increased the phosphorylation of AMPK and ACC in the liver tissues of HFD-fed mice. Based on the increase in the phosphorylation of AMPK and ACC by ALM16, we further investigated whether ALM16 downregulated lipid metabolism related to lipogenesis and lipid oxidation in the liver of HFD-fed mice. The results demonstrated that ALM16 treatment negatively regulated the lipogenesis-related proteins, including SREBP-1c and FAS, in the liver tissue of HFD-fed mice. It is generally recognized that the expression of lipogenesis-related proteins, such as SREBP-1c and FAS, are regulated by AMPK activation [12]. In the liver, SREBP-1c is an important transcription factor regulating fatty acid, cholesterol, and TG synthesis, and FAS is involved in lipid accumulation [57]. Previous studies have reported that downregulation of SREBP-1c and FAS, by the increased activation of AMPK, decreases lipid accumulation, and deposition, thereby improving hepatic steatosis [58, 59]. Our results showed that ALM16 reversed the HFD-induced downregulation of CPT-1, involved in fatty acid oxidation, in the HFD-fed mice. Similarly, several studies have reported that the protective effects against hepatic steatosis are mediated by the downregulation of genes involved in lipogenesis and upregulation genes associated with fatty acid oxidation via enhanced AMPK signaling [60, 61]. The results showed that AM treatment alone downregulated the FAS protein expression by activating AMPK phosphorylation, while it had no effect on the SREBP-1c and CPT-1 expressions. On the other hand, LE inhibited the expressions of SREBP-1c and FAS, while it had no effect on AMPK activation, suggesting that LE may act in a different way from AM, following other mechanisms where AMPK was not involved. Therefore, although the action mechanism between AM and LE underlying the observed positive effect of ALM16 on NAFLD is not fully understood at the moment, one of the possible explanations would be that it might be due to the cross-acting or complementary interaction between the constituent compounds in the two herb extracts, AM and LE, in ALM16, thereby resulting in a complementary multitarget effect on NAFLD. However, this preclinical study also has some limitations, including certain subjective biases due to the relatively small sample sizes, and some differences in features relevant to human NAFLD, which needs to be further clarified in the future.

Collectively, our results clearly demonstrated that ALM16 at a dose of 100 mg/kg could ameliorate NAFLD, through the downregulation of lipid metabolism-related proteins, especially modulating the AMPK and ACC signaling pathways. Although the detailed mechanism underlying the complementary interaction between the two herbs in ALM16 remains unknown, more potent effect on NAFLD was observed with ALM16 rather than individual extract (AM or LE) treatment.

## 5. Conclusions

The observed positive effects of ALM16 against hepatic steatosis and liver damage were, at least partly, shown to be mediated by the downregulation of SREBP-1c and FAS expression and upregulation of CPT-1 expression, through the modulation of AMPK and ACC signaling, in the established NAFLD *in vivo* model. Furthermore, this positive effect was more significant than the individual treatments with AM or LE. Although the clinical effectiveness of the observed hepatoprotective properties of ALM16 needs further investigation, these findings suggest that ALM16 could be used as a functional food supplement for the prevention or treatment of NAFLD.

## Data Availability

The data used to support the findings of this study are available from the corresponding author upon reasonable request.

## Supplementary Materials

Supplementary Materials Supplementary Figure 1: effects of ALM16 and individual extracts (AM and LE) on food intake (*n* = 6).
