# Supplementary material for: Protective Effect of a Mixture of Astragalus membranaceus and Lithospermum erythrorhizon Extract against Hepatic Steatosis in High Fat Diet-Induced Nonalcoholic Fatty Liver Disease Mice
Source: Evid Based Complement Alternat Med. 2020 Mar 19;2020:8370698. doi: 10.1155/2020/8370698 (PMC7106914; doi:10.1155/2020/8370698)
Supplement: Supplementary Materials — Supplementary Figure 1: effects of ALM16 and individual extracts (AM and LE) on food intake (n = 6). [file 8370698.f1.pdf]

Supplementary Figure 1.

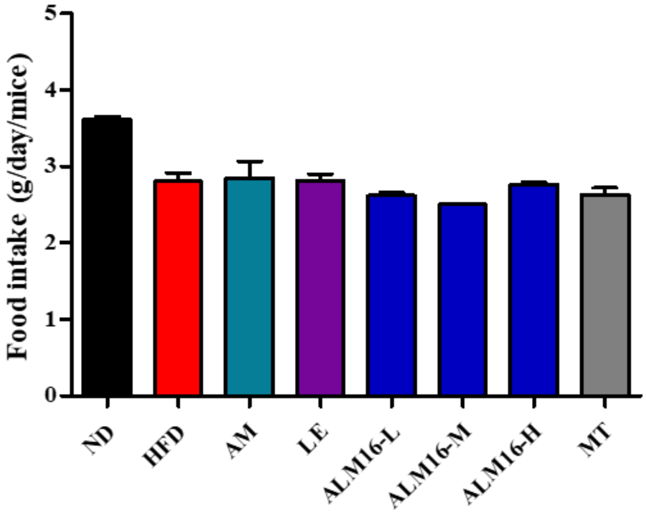

Supplementary Figure 1: Effects of ALM16 and individual extracts (AM and LE) on food intake (n=6). Data are expressed as mean ± standard error of the mean (SEM). $^{*}P < 0.05$, $^{**}P < 0.01$ and $^{***}P < 0.001$ vs HFD group. ND, normal diet; HFD, high fat diet; AM, HFD + AM extract (100 mg/kg); LE, HFD + LE extract (100 mg/kg); ALM16-L, HFD + ALM16 (50 mg/kg); ALM16-M, HFD + ALM16 (100 mg/kg); ALM16-H, HFD + ALM16 (200 mg/kg); MT, HFD + Milk thistle extract (100 mg/kg).
